# Polymorphisms in CCR5Δ32 and Risk of HIV-1 Infection in the Southeast of Caspian Sea, Iran

**DOI:** 10.1155/2017/4190107

**Published:** 2017-10-25

**Authors:** Zahra Heydarifard, Alijan Tabarraei, Abdolvahab Moradi

**Affiliations:** ^1^School of Medicine, Golestan University of Medical Sciences, Gorgan, Iran; ^2^Infectious Disease Research Center, Golestan University of Medical Sciences, Gorgan, Iran

## Abstract

Prevalence of CCR5Δ32 among blood samples of more than 400 healthy and HIV-1-infected people was investigated in Iran. Polymerase chain reaction (PCR) following DNA extraction was used. Desired frequency was analyzed by Hardy–Weinberg equilibrium (HWE) analysis and SPSS 16.0 software to harvest the results. The prevalence of CCRΔ32 heterozygote genotype was 3% in healthy people and 0.7% in HIV-1–infected individuals. There was no homozygote CCR5Δ32 in both groups, and the allele Δ32 was only observed in 1.5% and 0.36% of healthy and HIV-1–infected participants, respectively. Therefore according to this study, the frequency of the allele CCR5Δ32 indicates no significant difference between either groups (*p* = 0.18) and it sounds that the mentioned mutation in heterozygote people would not affect their susceptibility against HIV infection. Genotyping trial in Iranians with HIV infection is supposed to be helpful as a matter of prognostic purposes.

## 1. Introduction

Acquired immunodeficiency syndrome (AIDS) was initially diagnosed as an emerging disease in 1981. A retrovirus, named human immunodeficiency virus (HIV), is known to cause AIDS, and the lack of an efficient vaccine against it has made this virus as one of the most destructive complications in the world [1].

According to The Joint United Nations Programme on HIV/AIDS (UNAIDS), about 36.7 million people (34 to 39.8 million) have been suffering from HIV infection in the world by 2015. According to the recent Iranian official report, 28,663 individuals are infected with HIV while 6435 of them are struggling with AIDS. 1,800,000 people are living in Golestan Province, southeast of Caspian Sea. Of these, 194 people are suffering AIDS. Host genetic diversity has an important role in either disease susceptibility or resistance. However, the positive role of different genes in AIDS progression has still remained controversial [2].

HIV-1 isolates are categorized based on the chemokine receptors they recruit for entry. These isolates include R5, X4, and R5/X4 strains. R5 exclusively uses CCR5 coreceptors while X4 takes advantage of CXCR4 coreceptors and R5/X4 strains can use both [3].

CCR5 gene product, a chemokine receptor, is expressed on T cells, monocytes, macrophages, and dendritic cells. This is a specific receptor for the CC ligand 3 (CCL3), CCL4, and CCL5 chemokines as well as has an important role in the migration of immune cells to inflammatory sites [4, 5].

This protein is comprised of 352 amino acids (40.6 kD molecular weight) which form an N-terminal site, seven transmembrane domains, three extracellular loops, three intracellular loops, and a C-terminal domain. The N-terminal is the external site of the molecule and plays an important role in both R5 HIV interaction and chemokine binding process. The N-terminal is rich in tyrosine and acidic amino acids, and its coding gene is located on 3p21 chromosome along with groups of other chemokine coding genes such as CCR1, CCR2, CCR3, and XCR1 [6].

CXCR4 serves as a chemokine receptor for CXC chemokine ligand CXCL12 [also known as stromal cell-derived factor- (SDF-) 1 and pre-B-cell growth-stimulating factor (PBSF)], activates G proteins signaling transduction, and subsequently induces a rapid and transient rise in the level of intracellular calcium ions and chemotaxis [7, 8]. CXCL12/CXCR4 compound is vital for developmental processes, such as hematopoiesis, cardiogenesis, vascular formation, and neurogenesis as well as the maintenance of tissue stem cells [9].

Viral env glycoprotein complex, CD4 antigen, and the chemokine receptors CCR5 or CXCR4 located on the host cell surface are involved in HIV entry [10]. Coreceptor CCR5 plays the dominant role in HIV entry; however, there is a fact that indicates that the strain R5 has a significant contribution in HIV transition during the early phase of the viral life cycle, while the strain X4 emerges at the late phases of infection.

Deletions in CCR5 coding sequence, which result in missing of 32 base pairs of this gene (consisting of nucleotides 794 to 825), create a frameshift that leads to expression of seven novel amino acids (not existing in wild type) followed by an early stop codon at amino acid 182. Therefore, the truncated protein is no longer functional and will not be expressed on the host cell surface [6].

Patients with 32 bp truncation in CCR5 gene (CCR5Δ32) will not express CCR5 receptors on their cell surface and are highly protected against HIV-1 R5 strain infection; while on the other hand, this truncation will not affect the host's health status [11]. This polymorphism might have some kind of positive or negative relationship with other inflammatory diseases like systemic lupus erythematosus (SLE), rheumatoid arthritis (RA), and multiple sclerosis (MS) [12].

The allele CCR5Δ32 is not distributed in the world uniformly. Genetic studies have already identified that the prevalence of this variation among Caucasians is 10% [13], and in concurrence with this result, the molecular studies also have shown a rare distribution of this allele in HIV-1-infected patients and healthy individuals in Iran. Therefore, the average prevalence of this allele is thought to be 0.8% [13–16].

However, the prevalence of these genetic variants in Golestan Province, particularly in HIV-1-infected patients and healthy people with distinct ethnicities, is currently unknown. So, it would be of great urgency to determine how these genetic variants can contribute with people's susceptibility against HIV-1 R5 strain infection in the southeast of Caspian Sea.

## 2. Materials and Methods

### 2.1. Sample Collection

Three hundred blood samples were randomly collected from healthy people who had referred to Gorgan blood bank in Golestan Province, Iran, between December 2015 and February 2016. These samples were stored at −20°C along with one hundred forty blood samples which were simultaneously collected from HIV-1-infected individuals in the clinics of Golestan University of Medical Sciences, Iran. Essential questionnaire regarding age, sex, and CD4 counts was filled by the clinician. The detailed demographic parameters of both groups are presented in Table 1. Samples used in this study were checked for HIV-1 positivity by Western blot (WB) or real-time PCR.

### 2.2. Genomic DNA Extraction

Blood cells were pelleted at 13,000 rpm for 1 min. PBMCs were washed three to five times with TST buffer (10 mM Tris-HCl, pH 7.5, 5 mM MgCl_2_, 0.32 M sucrose, 1% Triton X-100) to remove erythrocytes. Then DNA was extracted from isolated PBMCs by KIAGEN DNA extraction kit (Kiagen, Tehran, Iran). The DNA purity was assessed with a spectrophotometer in A260nm/280 nm, and the products were kept at −20°C.

### 2.3. Genotyping

Polymerase chain reaction (PCR) was used for CCR5Δ32 genotyping in a total volume of 25 *μ*l. The reaction was performed with 50–100 ng/*μ*l genomic DNA, 10 pmol of forward and reverse primers, 0.3 mM dNTPs, 1.5 mM MgCl_2_, 1x PCR buffer (10 Mm Tris-HCl pH: 8/3, 50 Mm KCl, 1/5 MgCl_2_, and 0/001% (*w*/*v*) gelatin), and 1.5 unit Taq DNA polymerase. The amplification was done with an initial 5-minute step at 95°C, followed by 35 cycles, consisting of a denaturation step for 1 min at 95°C, an annealing step for 1 min at 62°C, and an elongation and final elongation step for 1 and 5 min at 72°C, respectively. Also, the sequence of primers used to detect Δ32 regions was 5′- CGTCTCTCCCAGGAATCATC-3′ for the forward primer and 5′- AGGGAGCCCAGAAGAGAAAA-3′ for the reverse one [12]. At the final stage, the wild type with a product of 276 bp was distinguished from CCR5Δ32 with a product of 244 bp on 3% gel electrophoresis.

### 2.4. Statistical Analysis

The distribution of the allele CCR5 was calculated by Hardy–Weinberg (HWN) equation (*a* + 2*A*)/2*N*, while *A* is the number of wild-type genotypes, *a* is the number of heterozygote genotypes, *N* is the number of total samples, and df (degree of freedom) is the subtraction of total genotypes and alleles. The frequency was further analyzed by Hardy–Weinberg equilibrium (HWE) analysis, and the differences in the frequency of each genetic variant between healthy and HIV-1-infected groups were determined by Chi-square or Fisher exact test, followed by analysis of the odds ratio and 95% confidence interval (CI). A value of *p* < 0.05 was considered statistically significant. The disease progression was determined based on changes in CD4+ cell counts of recruited patients.

### 2.5. Ethical Compliance

This work was approved by the Ethics Committee of Golestan University of Medical Sciences with the approval code IR.goums.REC.1394.252.

## 3. Results

**Figure 1:**
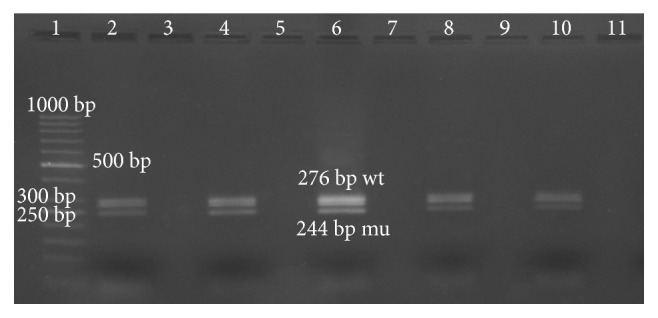
Electrophoresis migration patterns of CCR5Δ32 heterozygote in healthy group. Lane 1: 50 bp DNA ladder. Lane 2, 4, 6, 8, and 10 represent heterozygote wild type/Δ23 from healthy individuals. Lanes 3, 5, 7, 9, and 11 are empty.

**Figure 2:**
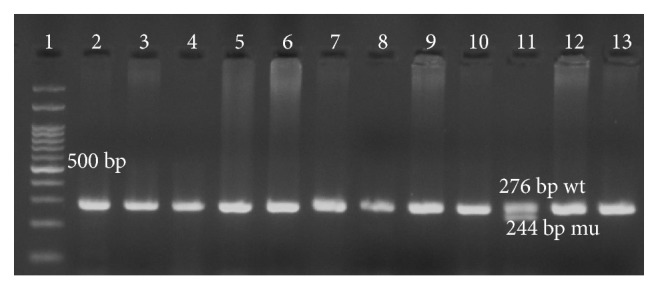
Electrophoresis migration patterns of CCR5 genotype in HIV group. Lane 1: 100 bp DNA ladder. Lane 2, 3, 4, 5, 6, 7, 8, 9, 10, 12, and 13 genotype wild type (wt/wt). Lane 11 represents heterozygote wild type/Δ23 from HIV individual.

Of three hundred individuals, nine (3%) were identified to have heterozygote Δ32 genotype, which implies the frequency of 1.5% (9/600) for this allele (Figure 1). Moreover, in the HIV group, only one of one hundred and forty samples was shown to have a heterozygote CCR5Δ32 genotype (Figure 2) and the frequency of this genotype and the allele Δ32 was 0.7% and 0.3%, respectively. CD4 count mean for a patient with heterozygous genotype was 322 cells/mm^3^, and it was not statistically significant (*p* = 0/48).

Of all samples that undergone genotyping process (four hundred and forty samples), the homozygote variant CCR5Δ32 was found none of the two groups and the frequency of allele CCR5Δ32 did not show a significant difference between healthy and HIV-1-infected people (*p* = 0.18) (Table 2).

Our data indicate that the frequency of mutation in CCR5Δ32 was low in different ethnicities and none of these ethnicities were found to be homozygous for the mentioned mutation (Table 3). In Turkmens, however, this mutation was more frequent in comparison with other ethnicities, but the difference was not of significance (*p* = 0.24). The total frequency of the wild-type allele (CCR5) and its mutant variant (CCR5Δ32) in the entire healthy samples was found to be 0.985 and 0.015, respectively. The frequency of deletions in CCR5Δ32 does not follow the Hardy–Weinberg equilibrium in the healthy and HIV-1-infected groups (*p* > 0.05, Chi-squared goodness of fit). Furthermore, the sequenced DNAs of CCR5Δ32 from Turkmens and Sistanians have been submitted to GenBank with the accession numbers KY025425 and KY025426, respectively.

## 4. Discussion

In the present study, the frequency of allele Δ32 in healthy people was found to be 1.5%, which indicates a higher frequency in comparison with HIV-1-infected patients (0.36%). We have investigated the frequency of CCR5Δ32 mutation among HIV-positive people and its relation to CD4 cell counts in the southeast of Caspian Sea, Iran. Our study did not find a relation between this mutation and HIV disease progression. Concurred to our finding, Wasik et al. in Poland [17], Deng et al. in China [18], Nkenfou et al. in Cameron [19], and Veloso et al. in Spain [20] have reported similar frequencies of Δ32 in healthy and HIV-1-infected persons. According to our study, there was no significant difference and subsequently no meaningful relationship in the frequency of allele Δ32 between healthy and HIV-1-infected groups. However, in contrast to our findings and the studies mentioned above, Tan et al. [21] have reported a higher frequency of Δ32 in HIV-1-seropositive people in comparison with seronegative individuals.

Besides in contrast to our study, Trecarichi et al. [22] have shown a significant higher frequency of Δ32 in the healthy control group (20%) in comparison with HIV-positive people (7.5%). Moreover, Philpott et al. [23] have reported a two-fold higher frequency of Δ32 genotype in the healthy control in comparison with HIV-1-seropositive people. But our findings show that the heterozygote and homozygote genotypes of Δ32 are present in only 3% and 0% of the healthy control group, respectively. This result is accompanied by previous studies in Iran including Gharagozloo et al. [13], Omrani [15], Rahimi et al. [14], and Arababadi et al. [24], which all have reported a frequency of less than 3% for such variations. This indicates that the distribution of heterozygote Δ32 has a low frequency in Iran. Here, based on our findings, we support the results of other studies previously done in Iran.

It has been reported that the allele Δ32 has a higher frequency in European Caucasian populations while the presence of this allele in Asian and African countries sounds rare. For instance, the frequency of this allele in the north of Europe, Italy, and Greece is reported 16%, 6%, and 4%, respectively [25]. However, more frequent distribution of this allele is observed in Sweden (14.2%) [26], Finland (26.6%) [27], Lithuania (25.9%) [28], and Estonia (18%) [29]. In Iran, based on the geographical characteristics, this frequency follows a different pattern and it seems to be higher in north and northwest of the country [14]. Therefore, since Golestan Province is already located in the southeast of Caspian Sea (north of Iran), it is supposed to show a higher rate of this polymorphism but due to the presence of different ethnicities living in this region (like Turkmen), the mutant genotype (CCR5Δ32) is more prevalent.

Considering this fact that there is a positive relationship between the prevalence of allele Δ32 and geographical factors [14, 30], we mostly expect to see a bigger distribution of this allele in the north of Iran. Additionally, diverse ethnicity in this region may be a positive factor in the presence of a more frequent heterozygote Δ32 genotype. Therefore, as the results show, there is a bigger distribution of heterozygote Δ32 genotype in the southeast of Caspian Sea, Iran.

Our study suggests that the role of ethnicity and race in CCR5Δ32 variants is more important in Turkmen since they showed a higher prevalence of this genotype (2.35%) in comparison with Sistanian (1.65%) and Fars (0.75%) groups; however, this difference was not of significance and further studies with a bigger sample size would be needed to verify these results in the future.

In multiple sclerosis (MS), CCR5 absence is supposed to play a role in reducing the migration of lymphocytes towards lesion sites and, therefore, it might suppress the pathogenesis of the disease and limit the inflammation of brain tissue. Shahbazi et al. have reported that the frequency of allele Δ32 in healthy and MS patients is 9% and 18%, respectively [31]. They also have concluded that the deletion of 32 nucleotides from CCR5 gene is associated with a higher risk of developing MS. These results seem to require further supporting investigations since it introduces a high prevalence of CCR5Δ32 in both healthy and patient people. Additionally, as Shahbazi et al., we have done the study on the population in the same region, whereas our study does not support such findings.

## 5. Conclusions

To the best of our knowledge and according to other studies, we expected to observe no heterozygote genotype for CCR5Δ32 in HIV-1-infected people, but surprisingly, our results showed a prevalence of 0.36% in Iranian HIV-1-positive patients who live in the southeast of Caspian Sea. It might be due to the presence of diverse ethnicities in this region; however, further studies with a bigger sample size might help to make the point clear.

## Figures and Tables

**Table 1:** Demographic characteristics of healthy individual and HIV-1-infected studied subjects.

Parameters	Healthy individual	HIV-1 infected
Age (median, range)	41 years (1–87)	38 years (7–59)
Number (%)		
Ethnicity		
Turkmen	106 (35.3)	
Sistanian	60 (20.0)	
Persian	134 (44.7)	140 (100%)
Gender		
Male	122 (40.7)	90 (64.3)
Female	178 (59.3)	50 (35.7)
Risk group		
Sexual contact		51 (36.4)
Intravenous drug user		69 (49.3)
Vertical transmission		5 (3.6)
Unknown		15 (10.7)
CD4 counts (cell/*μ*l)		
<200		69 (49.3)
201–349		41 (29.3)
350–499		13 (9.3)
>500		17 (12.1)
Total	300 (100)	140 (100)

**Table 2:** Allele frequency and Hardy–Weinberg equilibrium (HWE) analysis.

Group	Genotype of CCR5Δ32 (freq. %)			Allelic frequency (*λ*)		Δ32	Chi-squared	*p* value (1 df)
	Wt/wt	Wt/Δ32	Δ32/Δ32	*p* ^2^	*q* ^2^			
Healthy	291 (97%)	9 (3%)	0 (0%)	0.985	0.015	0.015	0.069	0.792
HIV-1-infected	139 (99.28%)	1 (0.72%)	0 (0%)	0.997	0.003	0.003	0.001	0.966

**Table 3:** Genotypic distribution and gene frequencies of the CCR5 allele in different ethnic population samples of Golestan region, southeast of Caspian Sea.

Group	Genotype of CCR5Δ32 (freq. %)			Allelic frequency (*λ*)		Δ32	Chi-squared	*p* value (1 df)
	Wt/wt	Wt/Δ32	Δ32/Δ32	*p* ^2^	*q* ^2^			
Turkmen	101 (95/3%)	5 (4/6%)	0 (0%)	0.976	0.023	0.023	0.061	0.803
Sistanian	58 (96.6%)	2 (3/2%)	0 (0%)	0.983	0.016	0.016	0.017	0.895
Persian	132 (98/5%)	2 (1/4%)	0 (0%)	0.992	0.007	0.007	0.007	0.93
